# Thiazole Analogues of the Marine Alkaloid Nortopsentin as Inhibitors of Bacterial Biofilm Formation

**DOI:** 10.3390/molecules26010081

**Published:** 2020-12-27

**Authors:** Anna Carbone, Stella Cascioferro, Barbara Parrino, Daniela Carbone, Camilla Pecoraro, Domenico Schillaci, Maria Grazia Cusimano, Girolamo Cirrincione, Patrizia Diana

**Affiliations:** Dipartimento di Scienze e Tecnologie Biologiche Chimiche e Farmaceutiche (STEBICEF), Università degli Studi di Palermo, Via Archirafi 32, 90123 Palermo, Italy; anna.carbone@unipa.it (A.C.); stellamaria.cascioferro@unipa.it (S.C.); barbara.parrino@unipa.it (B.P.); daniela.carbone@unipa.it (D.C.); camilla.pecoraro@unipa.it (C.P.); domenico.schillaci@unipa.it (D.S.); mariagrazia.cusimano@unipa.it (M.G.C.); girolamo.cirrincione@unipa.it (G.C.)

**Keywords:** antibiofilm agents, antibiotic resistance, thiazole derivatives, marine alkaloids analogues, nortopsentin

## Abstract

Anti-virulence strategy is currently considered a promising approach to overcome the global threat of the antibiotic resistance. Among different bacterial virulence factors, the biofilm formation is recognized as one of the most relevant. Considering the high and growing percentage of multi-drug resistant infections that are biofilm-mediated, new therapeutic agents capable of counteracting the formation of biofilms are urgently required. In this scenario, a new series of 18 thiazole derivatives was efficiently synthesized and evaluated for its ability to inhibit biofilm formation against the Gram-positive bacterial reference strains *Staphylococcus aureus* ATCC 25923 and *S. aureus* ATCC 6538 and the Gram-negative strain *Pseudomonas aeruginosa* ATCC 15442. Most of the new compounds showed a marked selectivity against the Gram-positive strains. Remarkably, five compounds exhibited BIC_50_ values against *S. aureus* ATCC 25923 ranging from 1.0 to 9.1 µM. The new compounds, affecting the biofilm formation without any interference on microbial growth, can be considered promising lead compounds for the development of a new class of anti-virulence agents.

## 1. Introduction

The development of synthetic small molecules able to counteract antibiotic resistance (AMR) mechanisms is urgently needed [1]. In fact, most antibiotics used to date to treat the most common infections are becoming ineffective. Many bacteria, including the well-known ESKAPE pathogens (*Enterococcus faecium*, *Staphylococcus aureus*, *Klebsiella pneumoniae*, *Acinetobacter baumannii*, *Pseudomonas aeruginosa*, and *Enterobacter* species) evolved in highly resistant forms through different mechanisms that include the inactivation of the antibiotic, chemical modification of the antibiotic target, alteration of cell permeability, and biofilm formation [2]. In particular, bacterial biofilm is currently considered one of the most relevant virulence factors, which is capable of making pathogens up to 1000 times more resistant than their planktonic form [3]. It was estimated that more than 80% of chronic infections are caused by biofilm formation on indwelling medical devices or host tissues [4].

Biofilm is a complex multicellular structure in which bacterial cells are embedded in a matrix constituted of extracellular polymeric substance (EPS), which is mainly formed by polysaccharides, proteins, lipids, extracellular DNA (e-DNA), and molecules originating from the host including mucus and DNA [5]. In the past decade, many efforts have been made for identifying new therapeutic strategy able to eradicate biofilm-associated infections, [6] and, despite numerous compounds being described as potent anti-biofilm agents, no new derivative has reached the clinic. The lack of approved anti-biofilm drugs together with the increase in the spread of chronic biofilm-related nosocomial infections make the research in this field particularly relevant.

Among the bioactive scaffolds recently described for their interesting anti-biofilm properties, thiazole derivatives are considered among the most promising compounds [7].

Sulfur-containing heterocycles are often involved in attractive nonbonding interactions that play an important role in the control of molecular conformation. In comparison with other five-membered heterocycles, the thiazole nucleus has unique features due to the presence of the low-lying C−S σ* orbitals. The small regions of low electron density present on the sulfur atom, known as σ-holes, are often involved in drug–target interactions, thus improving the affinity toward the biological receptor [8].

**Figure 1:**
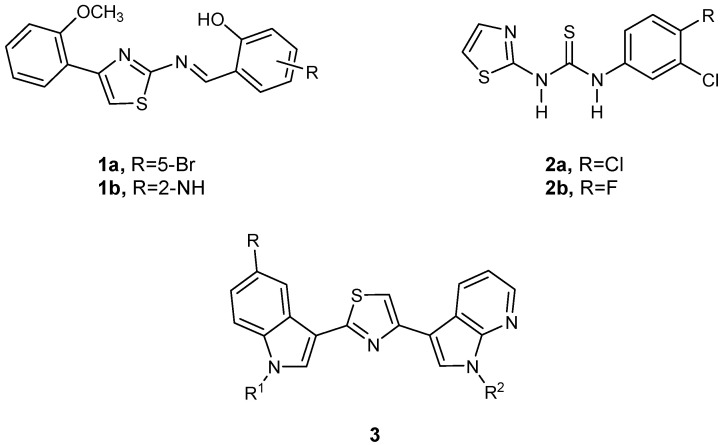
Chemical structures of the thiazole compounds **1a,b**; **2a,b**; and **3**.

Many thiazole compounds were reported in the last decade as potent anti-biofilm agents. The 4-(*o*-methoxyphenyl)-2-aminothiazoles **1a,b** (Figure 1) were found to be able to significantly inhibit *P. aeruginosa* biofilm formation at concentrations in the low micromolar range, interfering with the quorum sensing (QS) system [9]. The thiazole derivatives **2a,b** (Figure 1) showed potent anti-biofilm activity against eight methicillin-resistant (MRSE) and two reference (ATCC 12228, ATCC 35984) strains of *Staphylococcus epidermidis* eliciting BIC_50_ values ranging from 0.35 to 7.32 μg/mL [10].

On the basis of the interesting anti-biofilm properties described for the thiazole scaffold and continuing our search for new nortopsentin alkaloid analogues with promising biological activity [11,12,13], we recently reported the synthesis and the anti-biofilm activity of the new nortopsentin analogues of type **3**, in which the imidazole nucleus of the natural compound was replaced by the thiazole ring, and the indole moiety in position 4 was replaced by 7-azaindole (Figure 1) [14]. The thiazole derivatives **3** were tested against *S. aureus* ATCC 25923, *S. aureus* ATCC 6538, and *P. aeuruginosa* ATCC 15442 in order to evaluate their ability to inhibit biofilm formation and microbial growth. Most of the new thiazole nortopsentin analogues proved to be active as inhibitors of biofilm formation exhibiting marked selectivity toward staphylococcal biofilms, showing BIC_50_ values in the low micromolar range. Compounds of type **3** showed a typical anti-virulence profile, fighting bacterial virulence factors, such as biofilm formation, without interfering with the bacterial growth, thus imposing a low selective pressure for the onset of antibiotic resistance mechanisms.

With the aim to obtain more potent anti-biofilm agents that could be effective in the treatment of staphylococcal infections that are biofilm-mediated, herein, we report the synthesis of a new series of thiazole derivatives, structurally related to the nortopsentin analogues **3**, in which the 7-azaindole nucleus in position 4 of the thiazole ring was replaced by a thiophene (**1a–q**) or a pyridine ring (**2a–q**), and the aromatic bicyclic system in position 2 of the thiazole nucleus can be either an indole or a 7-azaindole moiety.

In fact, thiophene and pyridine moieties are recognized as valuable scaffolds in the development of potent anti-biofilm derivatives [15,16]. Additionally, the thiophene ring was recently discovered as a key nucleus in a series of compounds able to potently inhibit the virulence of relevant Gram-negative pathogens interfering with bacterial Disulfide bond enzyme A (DsbA) enzymes, which catalyzes disulfide bond formation in secreted and outer membrane proteins with virulence functions [17]. Therefore, since the so-far synthesized thiazole nortopsentin analogues have shown a strong selectivity toward the Gram-positive pathogens, we investigated whether the introduction of the thiophene ring could improve the anti-biofilm activity against the Gram-negative bacteria.

## 2. Results and Discussion

### 2.1. Chemistry

**Scheme 1:**
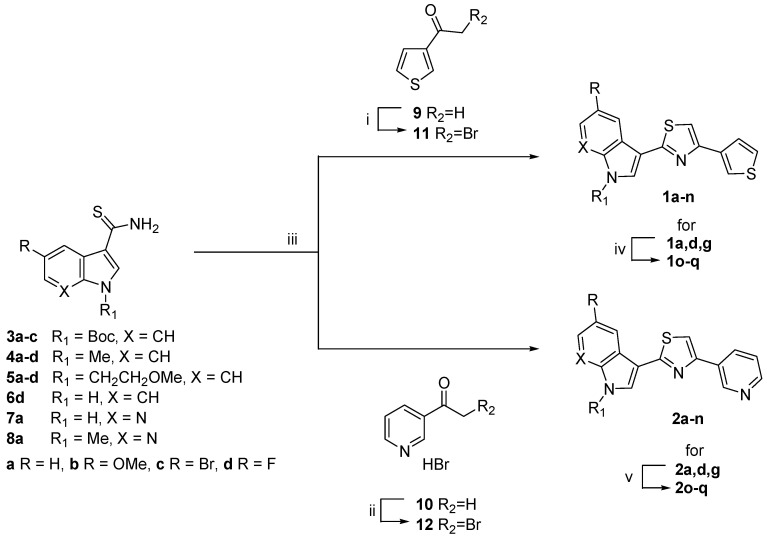
Synthesis of new thiazole derivatives of type **1a–q** and **2a–q**. Reagents: (i) (a) Br_2_, DCM, r.t., 1 h; (b) aq. NaHCO_3_, 80%; (ii) (a) 48% HBr, r.t. then 80 °C, Br_2_, 2 h; (b) acetone, 0 °C, 90%; (iii) EtOH, reflux, 30 min, 74–98% for **1a–n** and 73–99% for **2a–n**; (iv) (a) TFA, DCM, reflux, 24 h; (b) aq NaHCO_3_, 72–98%; (v) TFA, DCM, reflux, 24 h, 77–95%.

The new substituted thiazoles **1** and **2** (Scheme 1, Table 1 and Table 2) were efficiently synthesized by Hantzsch reaction between an appropriate thioamide of type **3–8**, previously obtained [18], and a suitable α-bromoacetyl derivative **11,12**.

The key intermediates **11,12** were prepared from the corresponding ethanones **9,10**. In particular, 1-thiophene-3-yl-ethanone **9** was converted into 2-bromo-1-thiophen-3-yl-ethanone **11** (80%), using bromine in dichloromethane [19]; while 1-pyridin-3-yl-ethanone **10** was turned into 2-bromo-1-pyridin-3-yl-ethanone hydrobromide **12** (90%) by reaction with 48% hydrobromic acid and bromine [20].

The reaction of thioamides **3a–c**, **4a–d**, **5a–d**, **6d**, **7a**, and **8a** with 2-bromo-1-heteroaryl-3-yl-ethanones **11** and **12** provided the desired 3-[4-(thiophen-3-yl)-1,3-thiazol-2-yl]-1*H*-indoles **1a–n** and 3-[4-(pyridin-3-yl)-1,3-thiazol-2-yl]-1*H*-indole hydrobromides **2a–n** in good to excellent yields (74–98% and 73–99%, respectively). The subsequent deprotection of *N*-tert-butylcarboxylate derivatives **1a**,**d**,**g** and **2a**,**d**,**g** using trifluoroacetic acid in dichloromethane under reflux, after neutralization with aqueous hydrogen carbonate solution, gave the thiazoles **1o–q** (72–98%), whereas thiazoles **2o–q** were directly isolated without neutralization (72–95%).

### 2.2. Biological Studies

All the new compounds were first tested for evaluating the antibacterial activity against the planktonic form of the Gram-positive *S. aureus* ATCC 25923, *S. aureus* ATCC 6538, and of the Gram-negative pathogen *P. aeruginosa* ATCC 15442. All the new thiazole derivatives, analogously to the precursors **3**, did not affect the microbial growth, showing Minimum Inhibitory Concentration (MIC) values greater than 100 µg/mL. This result is in agreement with the desired anti-virulence profile.

Inhibition of biofilm formation of the same bacterial strains was evaluated for all the new derivatives **1a–q** and **2a–q** at sub-MIC concentrations, and BIC_50_ values (the concentration of compound needed to inhibit biofilm formation by 50%) were determined for the compounds that showed a percentage of biofilm inhibition greater than 20% at the screening concentration of 10 µg/mL at least against one bacterial strain (Table 3).

**Figure 2:**
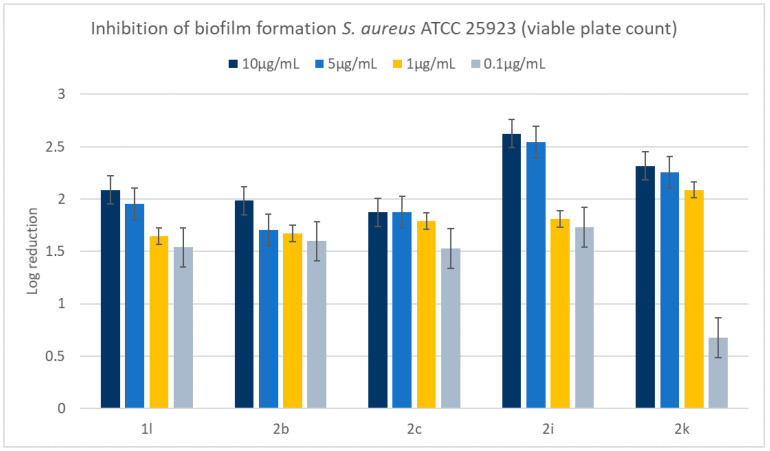
Inhibition of staphylococcal biofilm formation (viable plate count). Activity expressed in terms of log reduction. Each value is the average (reported with the SD values and error bars) from at least two independent experiments, *p* < 0.05.

All derivatives **1** and most of the compounds **2** showed antibiofilm activity, eliciting, as previously observed for the nortopsentin analogues of type **3**, a marked selectivity against the Gram-positive pathogens, in particular toward *S. aureus* ATCC 25923. Compounds **1l**, **2b**, **2c**, **2i**, and **2k** exhibited the highest potency with BIC_50_ values ranging from 1.0 to 9.1 µM. The replacement of the indole ring with the 7-azaindole moiety, as well as its substitution at position 5 with a halogen atom or a methoxy group, does not entail advantages in terms of the biofilm inhibition. Instead, the presence of a methoxyethyl group on the indole nitrogen generally led to an improvement of the antibiofilm activity against the staphylococcal strains. Compounds **1l**, **2b**, **2c**, **2i**, and **2k** were also tested by using viable plate count, and the activity of inhibition of staphylococcal biofilm formation was reported in terms of log reduction. By using such a method, compound **2i** was the most effective compound in interfering with biofilm formation, since it causes the greatest log reduction ranging from 2.62 to 1.73 at concentrations between 10 and 0.1 µg/mL (see Figure 2).

Most of the new thiazole derivatives **1** and **2** were inactive or weakly active against the Gram-negative strain. Only compounds **1a** and **2m** showed a significant inhibition of *P. aeruginosa* biofilm formation, eliciting BIC_50_ values of 14.9 and 5.5 µM, respectively.

Additionally, the most active compounds, for every bacterial strain, were selected and tested at the screening concentration of 100 μg/mL, for evaluating their dispersal activity against the 24 h preformed biofilm. No derivatives was able to interfere with the biofilm architecture; only compound **2m** showed weak dispersal activity eliciting a percentage of inhibition of 36% against *P. aeruginosa* at the screening concentration. Biological results highlighted the ability of the new compounds to interfere with the first stage of the biofilm life cycle, which consists in the bacterial adhesion to surfaces [21]. Anti-adhesion agents represent a valuable alternative to antibiotics, since they deprive the bacterium of its pathogenicity by preventing its adhesion to the host cells.

## 3. Materials and Methods

### 3.1. Chemistry

#### 3.1.1. General

All melting points were taken on a Büchi-Tottoly capillary apparatus (Büchi, Cornaredo, Italy) and are uncorrected. IR spectra were determined in bromoform with a Shimadzu FT/IR 8400S spectrophotometer (Shimadzu Corporation, Milan, Italy). ^1^H and ^13^C NMR spectra were measured at 200 and 50.0 MHz, respectively, in DMSO-*d*_6_ solution, using a Bruker Avance II series 200 MHz spectrometer (Bruker, Milan, Italy). Column chromatography was performed with Merk silica gel 230–400 mesh ASTM or with Büchi Sepacor chromatography module (prepacked cartridge system). Elemental analyses (C, H, and N) were within ± 0.4% of theoretical values. The purity of all the tested compounds was greater than 95%, as determined by HPLC (Agilent 1100 Series).

#### 3.1.2. General Procedures for the Synthesis of Thioamides **3a–c**, **4a–d**, **5a–d**, **6d**, **7a**, and **8a**

These compounds were prepared using procedures previously reported [17,18]. Analytical and spectroscopic data are in agreement with those previously reported.

#### 3.1.3. General Procedures for the Synthesis of 3-bromoacetyl compounds **11** and **12**

These compounds were prepared using known procedures (80–90%). Analytical and spectroscopic data are compatible with those previously reported [19,20].

#### 3.1.4. General Procedure for the Synthesis of Thiazole Derivatives (**1a–n**) and (**2a–n**)

A suspension of the proper thioamides **3a–c**, **4a–d**, **5a–d**, **6d**, **7a**, **8a** (2 mmol) and bromoacetyl derivatives **11, 12** (2 mmol) in anhydrous ethanol (8 mL) was refluxed for 30 min. After cooling, the obtained precipitate was filtered off, dried, and recrystallized from ethanol to give the desired thiazoles **1a–n** and **2a–n**.

Tert-Butyl-3-[4-(Thiophen-3-yl)-1,3-Thiazol-2-yl]-1H-Indole-1-Carboxylate (**1a**)

Yellow solid; yield: 79%; mp: 219–220 °C; IR cm^−1^: 1750 (CO); ^1^H NMR (200 MHz, DMSO-*d*_6_) δ: 1.68 (s, 9H, 3 x CH_3_), 7.40–7.53 (m, 2H, H-5′ and H-6′), 7.66–7.74 (m, 2H, H-4″ and H-5″), 8.00 (s, 1H, H-5), 8.10 (dd, 1H, *J* = 2.8, 1.3 Hz, H-2″), 8.16–8.20 (m, 1H, H-7′), 8.33 (s, 1H, H-2′), 8.47–8.52 (m, 1H, H-4′); ^13^C (50 MHz, DMSO-*d*_6_) δ: 27.6 (3 x q), 84.8 (s), 112.2 (d), 114.9 (d), 121.5 (d), 122.5 (d), 123.8 (d), 125.5 (d), 125.6 (d), 126.3 (d), 126.6 (s), 126.8 (s), 127.1 (d), 135.0 (s), 136.2 (s), 148.6 (s), 150.9 (s), 160.2 (s). Anal. Calcd for C_20_H_18_N_2_O_2_S_2_: C, 62.80; H, 4.74; N, 7.32%. Found: C, 62.59; H, 4.56; N, 7.21%.

1-Methyl-3-[4-(Thiophen-3-yl)-1,3-Thiazol-2-yl]-1H-Indole (**1b**)

Yellow solid; yield: 98%; mp: 248–249 °C; ^1^H NMR (200 MHz, DMSO-*d*_6_) δ: 3.89 (s, 3H, CH_3_), 7.23–7.36 (m, 2H, H-5′ and H-6′), 7.55–7.72 (m, 3H, H-4″, H-5″ and H-7′), 7.79 (s, 1H, H-5), 8.03 (dd, 1H, *J* = 2.8, 1.3 Hz, H-2″), 8.19 (s, 1H, H-2′), 8.31–8.35 (m, 1H, H-4′); ^13^C (50 MHz, DMSO-*d*_6_) δ: 32.9 (q), 109.1 (s), 110.0 (d), 110.6 (d), 120.6 (d), 121.1 (d), 122.2 (d), 122.5 (d), 124.5 (s), 126.3 (d), 127.0 (d), 130.8 (d), 136.2 (s), 137.0 (s), 149.8 (s), 162.5 (s). Anal. Calcd for C_16_H_12_N_2_S_2_: C, 64.83; H, 4.08; N, 9.45%. Found: C, 65.11; H, 3.91; N, 9.59%.

1-(2-Methoxyethyl)-3-[4-(Thiophen-3-yl)-1,3-Thiazol-2-yl]-1H-Indole (**1c**)

Orange solid; yield: 80%; mp: 169–170 °C; ^1^H NMR (200 MHz, DMSO-*d*_6_) δ: 3.24 (s, 3H, CH_3_), 3.72 (t, 2H, *J* = 5.1 Hz, CH_2_), 4.46 (t, 2H, *J* = 5.1 Hz, CH_2_), 7.23–7.33 (m, 2H, H-5′ and H-6′), 7.62–7.81 (m, 3H, H-4″, H-5″ and H-7′), 7.81 (s, 1H, H-5), 8.04 (dd, 1H, *J* = 2.8, 1.4 Hz, H-2″), 8.17 (s, 1H, H-2′), 8.30–8.34 (m, 1H, H-4′); ^13^C (50 MHz, DMSO-*d*_6_) δ: 45.7 (t), 58.1 (q), 70.6 (t), 109.3 (s), 110.1 (d), 110.9 (d), 120.5 (d), 121.1 (d), 122.3 (d), 122.5 (d), 124.6 (s), 126.3 (d), 127.0 (d), 130.3 (d), 136.1 (s), 136.5 (s), 149.7 (s), 162.4 (s). Anal. Calcd for C_18_H_16_N_2_OS_2_: C, 63.50; H, 4.74; N, 8.23%. Found: C, 63.26; H, 4.90; N, 8.02%.

Tert-Butyl 5-Methoxy-3-[4-(Thiophen-3-yl)-1,3-Thiazol-2-yl]-1H-Indole-1-Carboxylate (**1d**)

Pink solid; yield: 89%; mp: 185–186 °C; IR cm^−1^: 1733 (CO); ^1^H NMR (200 MHz, DMSO-*d*_6_) δ: 1.67 (s, 9H, 3 x CH_3_), 3.90 (s, 3H, CH_3_), 7.09 (dd, 1H, *J* = 9.0, 2.6 Hz, H-6′), 7.66–7.73 (m, 2H, H-4″ and H-7′), 7.98 (s, 1H, H-5), 8.04–8.07 (m, 3H, H-2″, H-4′ and H-5″), 8.29 (s, 1H, H-2′); ^13^C (50 MHz, DMSO-*d*_6_) δ: 27.6 (3 x q), 55.3 (q), 84.7 (s), 103.8 (d), 112.2 (d), 114.2 (d), 114.7 (s), 115.8 (d), 122.4 (d), 126.1 (d), 126.2 (d), 127.2 (d), 127.6 (s), 129.6 (s), 136.2 (s), 148.6 (s), 150.8 (s), 156.2 (s), 160.4 (s). Anal. Calcd for C_21_H_20_N_2_O_3_S_2_: C, 61.14; H, 4.89; N, 6.79%. Found: C, 60.89; H, 5.03; N, 6.56%.

5-Methoxy-1-Methyl-3-[4-(Thiophen-3-yl)-1,3-Thiazol-2-yl]-1H-Indole (**1e**)

Orange solid; yield: 98%; mp: 232–233 °C; ^1^H NMR (200 MHz, DMSO-*d*_6_) δ: 3.86 (s, 3H, CH_3_), 3.87 (s, 3H, CH_3_), 6.95 (dd, 1H, *J* = 8.9, 2.5 Hz, H-6′), 7.48 (d, 1H, *J* = 8.9 Hz, H-7′), 7.66–7.69 (m, 2H, H-4″ and H-5″), 7.77 (s, 1H, H-5), 7.83 (d, 1H, *J* = 2.5 Hz, H-4′), 8.00 (dd, 1H, *J* = 2.7, 1.5 Hz, H-2″), 8.13 (s, 1H, H-2′); ^13^C (50 MHz, DMSO-*d*_6_) δ: 33.1 (q), 55.3 (q), 102.3 (d), 108.5 (s), 109.8 (d), 111.5 (d), 112.4 (d), 122.2 (d), 125.1 (s), 126.2 (d), 127.1 (d), 131.2 (d), 132.2 (s), 136.0 (s), 149.5 (s), 155.0 (s), 162.7 (s). Anal. Calcd for C_17_H_14_N_2_OS_2_: C, 62.55; H, 4.32; N, 8.58%. Found: C, 62.31; H, 4.15; N, 8.32%.

5-Methoxy-1-(2-Methoxyethyl)-3-[4-(Thiophen-3-yl)-1,3-Thiazol-2-yl]-1H-Indole (**1f**)

Yellow solid; yield: 91%; mp: 213–214 °C; ^1^H NMR (200 MHz, DMSO-*d*_6_) δ: 3.24 (s, 3H, CH_3_), 3.69 (t, 2H, *J* = 5.1 Hz, CH_2_), 3.87 (s, 3H, CH_3_), 4.41 (t, 2H, *J* = 5.1 Hz, CH_2_), 6.93 (dd, 1H, *J* = 8.9, 2.5 Hz, H-6′), 7.55 (d, 1H, *J* = 8.9 Hz, H-7′), 7.64–7.71 (m, 2H, H-4″ and H-5″), 7.78 (s, 1H, H-5), 7.83 (d, 1H, *J* = 2.5 Hz, H-4′), 8.00 (dd, 1H, *J* = 2.7, 1.5 Hz, H-2″), 8.10 (s, 1H, H-2′); ^13^C (50 MHz, DMSO-*d*_6_) δ: 45.9 (t), 55.3 (q), 58.1 (q), 70.7 (t), 102.3 (d), 108.8 (s), 109.8 (d), 111.8 (d), 112.3 (d), 121.2 (d), 125.1 (s), 126.2 (d), 127.1 (d), 130.6 (d), 131.6 (s), 136.1 (s), 149.5 (s), 154.9 (s), 162.6 (s). Anal. Calcd for C_19_H_18_N_2_O_2_S_2_: C, 61.60; H, 4.90; N, 7.56%. Found: C, 61.45; H, 4.79; N, 7.39%.

Tert-Butyl 5-Bromo-3-[4-(Thiophen-3-yl)-1,3-Thiazol-2-yl]-1H-Indole-1-Carboxylate (**1g**)

Pink solid; yield: 85%; mp: 174–175 °C; IR cm−^1^: 1739 (CO); ^1^H NMR (200 MHz, DMSO-*d*_6_) δ: 1.68 (s, 9H, 3 x CH_3_), 7.64 (dd, 1H, *J* = 8.9, 2.0 Hz, H-6′), 7.70–7.71 (m, 2H, H-4″ and H-5″), 8.01 (s, 1H, H-5), 8.04 (dd, 1H, *J* = 2.1, 1.3 Hz, H-2″), 8.11 (d, 1H, *J* = 8.9 Hz, H-7′), 8.38 (s, 1H, H-2′), 8.61 (d, 1H, *J* = 2.0 Hz, H-4′); ^13^C (50 MHz, DMSO-*d*_6_) δ: 27.6 (3 x q), 85.4 (s), 112.6 (d), 116.4 (s), 116.9 (d), 119.3 (d), 122.4 (d), 123.8 (d), 126.2 (d), 127.0 (d), 127.3 (s), 128.1 (d), 128.4 (s), 133.9 (s), 136.1 (s), 148.3 (s), 150.9 (s), 159.8 (s). Anal. Calcd for C_20_H_17_BrN_2_O_2_S_2_: C, 52.06; H, 3.71; N, 6.07%. Found: C, 51.85; H, 3.94; N, 6.22%.

5-Bromo-1-Methyl-3-[4-(Thiophen-3-yl)-1,3-Thiazol-2-yl]-1H-Indole (**1h**)

Orange solid; yield: 90%; mp: 250–251 °C; ^1^H NMR (200 MHz, DMSO-*d*_6_) δ: 3.89 (s, 3H, CH_3_), 7.44 (dd, 1H, *J* = 8.7, 1.9 Hz, H-6′), 7.58 (d, 1H, *J* = 8.7 Hz, H-7′), 7.67–7.68 (m, 2H, H-4″ and H-5″), 7.81 (s, 1H, H-5), 7.99 (dd, 1H, *J* = 2.1, 1.3 Hz, H-2″), 8.24 (s, 1H, H-2′), 8.47 (d, 1H, *J* = 1.9 Hz, H-4′); ^13^C (50 MHz, DMSO-*d*_6_) δ: 33.1 (q), 108.8 (s), 110.3 (d), 112.8 (d), 113.8 (s), 122.1 (d), 122.8 (d), 125.0 (d), 126.1 (s), 126.2 (d), 127.1 (d), 132.0 (d), 135.8 (s), 136.4 (s), 150.3 (s), 161.8 (s). Anal. Calcd for C_16_H_11_BrN_2_S_2_: C, 51.20; H, 2.95; N, 7.46%. Found: C, 50.93; H, 2.90; N, 7.18%.

5-Bromo-1-(2-Methoxyethyl)-3-[4-(Thiophen-3-yl)-1,3-Thiazol-2-yl]-1H-Indole (**1i**)

Yellow solid; yield: 92%; mp: 226–227 °C; ^1^H NMR (200 MHz, DMSO-*d*_6_) δ: 3.24 (s, 3H, CH_3_), 3.72 (t, 2H, *J* = 5.0 Hz, CH_2_), 4.46 (t, 2H, *J* = 5.0 Hz, CH_2_), 7.42 (dd, 1H, *J* = 8.8, 2.0 Hz, H-6′), 7.65 (d, 1H, *J* = 8.8 Hz, H-7′), 7.63–7.68 (m, 2H, H-4″ and H-5″), 7.81 (s, 1H, H-5), 7.99 (dd, 1H, *J* = 2.1, 1.3 Hz, H-2″), 8.22 (s, 1H, H-2′), 8.46 (d, 1H, *J* = 2.0 Hz, H-4′); ^13^C (50 MHz, DMSO-*d*_6_) δ: 46.0 (t), 58.1 (q), 70.6 (t), 109.0 (s), 110.4 (d), 113.1 (d), 113.8 (s), 122.1 (d), 122.7 (d), 125.0 (d), 126.1 (s), 126.2 (d), 127.1 (d), 131.5 (d), 135.4 (s), 136.3 (s), 150.2 (s), 161.8 (s). Anal. Calcd for C_18_H_15_BrN_2_OS_2_: C, 51.55; H, 3.61; N, 6.68%. Found: C, 51.27; H, 3.77; N, 6.81%.

5-Fluoro-3-[4-(Thiophen-3-yl)-1,3-Thiazol-2-yl]-1H-Indole (**1j**)

Yellow solid; yield: 74%; mp: 209–210 °C; IR cm^−1^: 3270 (NH); ^1^H NMR (200 MHz, DMSO-*d*_6_) δ: 7.10 (td, 1H, *J* = 11.6, 9.2, 2.6 Hz, H-6′), 7.51 (dd, 1H, *J* = 11.6, 4.6 Hz, H-7′), 7.64–7.71 (m, 2H, H-4″ and H-5″), 7.79 (s, 1H, H-5), 8.01–8.08 (m, 2H, H-2″ and H-4′), 8.23 (d, 1H, *J* = 2.9 Hz, H-2′); 11.90 (bs, 1H, NH); ^13^C (50 MHz, DMSO-*d*_6_) δ: 105.3 (d, *J_C6′-F_* = 24.4 Hz), 110.0 (d), 110.4 (d, *J_C7′a-F_* = 4.6 Hz), 110.2 (d, *J_C4′F_* = 26.0 Hz), 113.4 (d, *J_C7′-F_* = 9.6 Hz), 122.2 (d), 124.5 (d, *J_C3′a-F_* = 10.8 Hz), 126.2 (d), 127.0 (d), 128.7 (d), 133.2 (s), 134.3 (s), 136.4 (s), 150.1 (s), 157.9 (d, *J_C5′-F_* = 235 Hz). Anal. Calcd for C_15_H_9_FN_2_S_2_: C, 59.98; H, 3.02; N, 9.33%. Found: C, 59.67; H, 2.93; N, 9.18%.

5-Fluoro-1-Methyl-3-[4-(Thiophen-3-yl)-1,3-Thiazol-2-yl]-1H-Indole (**1k**)

Yellow solid; yield: 95%; mp: 231–232 °C; ^1^H NMR (200 MHz, DMSO-*d*_6_) δ: 3.90 (s, 3H, CH_3_), 7.18 (td, 1H, *J* = 11.7, 9.2, 2.6 Hz, H-6′), 7.57–7.71 (m, 3H, H-4″, H-5″ and H-7′), 7.78 (s, 1H, H-5), 8.02–8.08 (m, 2H, H-2″ and H-4′), 8.25 (s, 1H, H-2′); ^13^C (50 MHz, DMSO-*d*_6_) δ: 33.2 (q), 105.5 (d, *J_C6′-F_* = 24.4 Hz), 109.3 (d, *J_C7′a-F_* = 4.6 Hz), 110.0 (d), 110.7 (d, *J_C4′F_* = 26.3 Hz), 113.5 (d, *J_C7′-F_* = 9.7 Hz), 122.6 (d), 124.8 (d, *J_C3′a-F_* = 10.8 Hz), 126.2 (d), 127.0 (d), 132.4 (d), 133.8 (s), 136.3 (s), 150.2 (s), 157.2 (d, *J_C5′-F_* = 234 Hz), 162.1 (s). Anal. Calcd for C_16_H_11_FN_2_S_2_: C, 61.12; H, 3.53; N, 8.91%. Found: C, 60.88; H, 3.47; N, 8.74%.

5-Fluoro-1-(2-Methoxyethyl)-3-[4-(Thiophen-3-yl)-1,3-Thiazol-2-yl]-1H-Indole (**1l**)

Yellow solid; yield: 88%; mp: 200–201 °C; ^1^H NMR (200 MHz, DMSO-*d*_6_) δ: 3.25 (s, 3H, CH_3_), 3.72 (t, 2H, *J* = 5.0 Hz, CH_2_), 4.46 (t, 2H, *J* = 5.0 Hz, CH_2_), 7.16 (td, 1H, *J* = 11.8, 9.2, 2.6 Hz, H-6′), 7.64–7.71 (m, 3H, H-4″, H-5″ and H-7′), 7.80 (s, 1H, H-5), 8.01–8.07 (m, 2H, H-2″ and H-4′), 8.23 (s, 1H, H-2′); ^13^C (50 MHz, DMSO-*d*_6_) δ: 46.0 (t), 58.1 (q), 70.7 (t), 105.5 (d, *J_C6′-F_* = 25.0 Hz), 109.5 (d, *J_C7′a-F_* = 4.6 Hz), 110.1 (d), 110.7 (d, *J_C4′F_* = 26.1 Hz), 117.3 (d, *J_C7′-F_* = 9.8 Hz), 122.3 (d), 124.9 (d, *J_C3′a-F_* = 10.7 Hz), 126.2 (d), 127.0 (d), 131.8 (d), 133.3 (s), 136.3 (s), 150.2 (s), 158.1 (d, *J_C5′-F_* = 234 Hz), 162.0 (s). Anal. Calcd for C_18_H_15_FN_2_OS_2_: C, 60.31; H, 4.22; N, 7.82%. Found: C, 60.03; H, 4.05; N, 7.64%.

3-[4-(Thiophen-3-yl)-1,3-Thiazol-2-yl]-1H-Pyrrolo[2,3-b]Pyridine (**1m**)

Yellow solid; yield: 93%; mp: 268–269 °C; IR cm^−1^: 3273 (NH); ^1^H NMR (200 MHz, DMSO-*d*_6_) δ: 7.37 (dd, 1H, *J* = 7.9, 4.9 Hz, H-5′), 7.64–7.73 (m, 2H, H-4″ and H-5″), 7.84 (s, 1H, H-5), 8.09 (dd, 1H, *J* = 2.8, 1.3 Hz, H-2″), 8.34 (d, 1H, *J* = 2.0 Hz, H-2′), 8.41 (dd, 1H, *J* = 4.9, 1.5 Hz, H-6′), 8.80 (dd, 1H, *J* = 7.9, 1.5 Hz, H-4′), 12.50 (bs, 1H, NH); ^13^C (50 MHz, DMSO-*d*_6_) δ: 111.0 (s), 112.1 (d), 118.2 (d), 120.2 (s), 123.4 (d), 127.2 (d), 128.0 (d), 129.2 (d), 134.4 (d), 137.2 (s), 141.0 (d), 145.2 (s), 151.4 (s), 162.1 (s). Anal. Calcd for C_14_H_9_N_3_S_2_: C, 59.34; H, 3.20; N, 14.83%. Found: C, 59.03; H, 3.44; N, 15.06%.

1-Methyl-3-[4-(Thiophen-3-yl)-1,3-Thiazol-2-yl]-1H-Pyrrolo[2,3-b]Pyridine (**1n**)

Yellow solid; yield: 96%; mp: 254–255 °C; ^1^H NMR (200 MHz, DMSO-*d*_6_) δ: 3.92 (s, 3H, CH_3_), 7.34 (dd, 1H, *J* = 7.9, 4.7 Hz, H-5′), 7.64–7.73 (m, 2H, H-4″ and H-5″), 7.83 (s, 1H, H-5), 8.07 (dd, 1H, *J* = 2.8, 1.3 Hz, H-2″), 8.38 (s, 1H, H-2′), 8.42 (dd, 1H, *J* = 4.7, 1.6 Hz, H-6′), 8.72 (dd, 1H, *J* = 7.9, 1.6 Hz, H-4′); ^13^C (50 MHz, DMSO-*d*_6_) δ: 31.7 (q), 108.3 (s), 110.7 (d), 117.3 (d), 118.0 (s), 122.4 (d), 126.3 (d), 127.0 (d), 131.1 (2 x d), 136.3 (s), 142.2 (d), 146.0 (s), 150.4 (s), 161.3 (s). Anal. Calcd for C_15_H_11_N_3_S_2_: C, 60.58; H, 3.73; N, 14.13%. Found: C, C, 60.33; H, 3.59; N, 13.89%.

Tert-Butyl 3-[4-(Pyridin-3-yl)-1,3-Thiazol-2-yl]-1H-Indole-1-Carboxylate Hydrobromide (**2a**)

Pink solid; yield: 90%; mp: 171–172 °C; IR cm^−1^: 1737 (CO), 3420 (NH^+^); ^1^H NMR (200 MHz, DMSO-*d*_6_) δ: 1.69 (s, 9H, 3 x CH_3_), 7.43–7.54 (m, 2H, H-5′ and H-6′), 8.09 (dd, 1H, *J* = 8.1, 5.4 Hz, H-5″), 8.14–8.23 (m, 1H, H-7′), 8.44 (s, 1H, H-2′), 8.49–8.53 (m, 1H, H-4′), 8.62 (s, 1H, H-5), 8.88 (d, 1H, *J* = 5.4 Hz, H-6″), 9.08 (d, 1H, *J* = 8.1 Hz, H-4″), 9.53 (s, 1H, H-2″); ^13^C (50 MHz, DMSO-*d*_6_) δ: 27.6 (3xq), 85.0 (s), 114.3 (s), 115.0 (d), 118.1 (d), 121.5 (d), 124.0 (d), 125.6 (d), 126.3 (d), 126.5 (s), 127.2 (d), 132.4 (s), 134.9 (s), 140.1 (d), 141.3 (d), 141.6 (d), 148.4 (s), 148.5 (s), 161.8 (s). Anal. Calcd for C_21_H_20_BrN_3_O_2_S: C, 55.03; H, 4.40; N, 9.17%. Found: C, 54.78; H, 4.57; N, 8.98%.

1-Methyl-3-[4-(Pyridin-3-yl)-1,3-Thiazol-2-yl]-1H-Indole Hydrobromide (**2b**)

Orange solid; yield: 79%; mp: 266–267 °C; IR cm^−1^ 3423 (NH^+^); ^1^H NMR (200 MHz, DMSO-*d*_6_) δ: 3.91 (s, 3H, CH_3_), 7.25–7.38 (m, 2H, H-5′ and H-6′), 7.57–7.61 (m, 1H, H-7′), 8.21 (dd, 1H, *J* = 8.2, 5.7 Hz, H-5″), 8.29 (s, 1H, H-2′), 8.35–8.40 (m, 1H, H-4′), 8.52 (s, 1H, H-5), 8.94 (d, 1H, *J* = 5.7 Hz, H-6″), 9.21 (d, 1H, *J* = 8.2 Hz, H-4″), 9.55 (d, 1H, *J* = 1.7 Hz, H-2″); ^13^C (50 MHz, DMSO-*d*_6_) δ: 32.9 (q); 108.8 (s), 110.7 (d), 116.2 (d), 120.6 (d), 121.3 (d), 122.7 (d), 124.4 (s), 127.5 (d), 131.4 (d), 133.1 (s), 137.0 (s), 139.0 (d), 140.3 (d), 142.1 (d), 147.5 (s), 163.9 (s). Anal. Calcd for: C_17_H_14_BrN_3_S: C, 54.85; H, 3.79; N, 11.29%. Found: C, 54.64; H, 4.00; N, 11.47%.

1-(2-Methoxyethyl)-3-[4-(Pyridin-3-yl)-1,3-Thiazol-2-yl]-1H-Indole Hydrobromide (**2c**)

Orange solid; yield: 73%; mp: 190–191 °C; IR cm^−1^ 3425 (NH^+^); ^1^H NMR (200 MHz, DMSO-*d*_6_) δ: 3.24 (s, 3H, CH_3_), 3.73 (t, 2H, *J* = 5.0 Hz, CH_2_), 4.47 (t, 2H, *J* = 5.0 Hz, CH_2_), 7.25–7.35 (m, 2H, H-5′ and H-6′), 7.64–7.68 (m, 1H, H-7′), 8.23 (dd, 1H, *J* = 8.2, 5.8 Hz, H-5″), 8.27 (s, 1H, H-2′), 8.33–8.37 (m, 1H, H-4′), 8.56 (s, 1H, H-5), 8.82 (d, 1H, *J* = 5.8 Hz, H-6″), 9.24 (d, 1H, *J* = 8.2 Hz, H-4″), 9.58 (d, 1H, *J* = 1.5 Hz, H-2″); ^13^C (50 MHz, DMSO-*d*_6_) δ: 45.8 (t), 50.1 (q), 70.6 (t), 109.1 (s), 111.0 (d), 116.3 (d), 120.6 (d), 121.3 (d), 122.7 (d), 124.5 (s), 127.6 (d), 131.0 (d), 133.1 (s), 136.6 (s), 139.1 (d), 140.4 (d), 142.3 (d), 147.5 (s), 163.9 (s). Anal. Calcd for: C_19_H_18_BrN_3_OS: C, 54.81; H, 4.36; N, 10.09%. Found: C, 54.60; H, 4.21; N, 9.92%.

Tert-Butyl 5-Methoxy-3-[4-(Pyridin-3-yl)-1,3-Thiazol-2-yl]-1H-Indole-1-Carboxylate Hydrobromide (**2d**)

Orange solid; yield: 88%; mp: 154–155 °C; IR cm^−1^: 1734 (CO), 3422 (NH^+^); ^1^H NMR (200 MHz, DMSO-*d*_6_) δ: 1.68 (s, 9H, 3 x CH_3_), 3.91 (s, 3H, CH_3_), 7.11 (dd, 1H, *J* = 9.1, 2.6 Hz, H-6′), 7.82 (d, 1H, *J* = 2.6 Hz, H-4′), 8.05 (d, 1H, *J* = 9.1 Hz, H-7′), 8.13 (dd, 1H, *J* = 8.2, 5.6 Hz, H-5″), 8.38 (s, 1H, H-2′), 8.63 (s, 1H, H-5), 8.91 (d, 1H, *J* = 5.6 Hz, H-6″), 9.07 (d, 1H, *J* = 8.2 Hz, H-4″), 9.50 (d, 1H, *J* = 2.0 Hz, H-2″); ^13^C (50 MHz, DMSO-*d*_6_) δ: 27.6 (3 x q), 55.4 (q), 84.9 (s), 103.8 (d), 114.0 (d), 115.8 (d), 118.0 (d), 126.9 (d), 127.2 (d), 129.5 (s), 132.4 (s), 140.0 (d), 141.2 (d), 141.3 (s), 141.5 (d), 148.2 (s), 148.5 (s), 149.7 (s), 156.2 (s), 161.9 (s). Anal. Calcd for: C_22_H_22_BrN_3_O_3_S: C, 54.10; H, 4.54; N, 8.60%. Found: C, 54.35; H, 4.30; N, 8.46%.

5-Methoxy-1-Methyl-3-[4-(Pyridin-3-yl)-1,3-Thiazol-2-yl]-1H-Indole Hydrobromide (**2e**)

Orange solid; yield: 96%; mp: 210–211 °C; IR cm^−1^ 3429 (NH^+^); ^1^H NMR (200 MHz, DMSO-*d*_6_) δ: 3.87 (s, 3H, CH_3_), 3.90 (s, 3H, CH_3_), 6.99 (dd, 1H, *J* = 8.9, 2.4 Hz, H-6′), 7.63 (d, 1H, *J* = 8.9 Hz, H-7′), 7.83 (d, 1H, *J* = 2.4 Hz, H-4′), 8.19–8.25 (m, 2H, H-2′ and H-5″), 8.50 (s, 1H, H-5), 8.94 (d, 1H, *J* = 5.3 Hz, H-6″), 9.20 (d, 1H, *J* = 8.3 Hz, H-4″), 9.51 (s, 1H, H-2″); ^13^C (50 MHz, DMSO-*d*_6_) δ: 33.1 (q), 55.4 (q), 102.5 (d), 108.4 (s), 111.6 (d), 112.3 (d), 115.9 (d), 125.0 (s), 127.5 (d), 131.7 (d), 132.2 (s), 133.1 (s), 139.1 (d), 140.4 (d), 141.9 (d), 147.4 (s), 155.1 (s), 164.2 (s). Anal. Calcd for: C_18_H_16_BrN_3_OS: C, 53.74; H, 4.01; N, 10.44%. Found: C, 53.50; H, 3.83; N, 10.70%.

5-Methoxy-1-(2-Methoxyethyl)-3-[4-(Pyridin-3-yl)-1,3-Thiazol-2-yl]-1H-Indole Hydrobromide (**2f**)

Orange solid; yield: 89%; mp: 122–123 °C; IR cm^−1^ 3426 (NH^+^); ^1^H NMR (200 MHz, DMSO-d_6_) δ: 3.24 (s, 3H, CH_3_), 3.71 (t, 2H, *J* = 5.0 Hz, CH_2_), 3.89 (s, 3H, CH_3_), 4.42 (t, 2H, *J* = 5.0 Hz, CH_2_), 6.95 (dd, 1H, *J* = 8.9, 2.5 Hz, H-6′), 7.56 (d, 1H, *J* = 8.9 Hz, H-7′), 7.81 (d, 1H, *J* = 2.5 Hz, H-4′), 8.18–8.22 (m, 2H, H-2′ and H-5″), 8.50 (s, 1H, H-5), 8.93 (d, 1H, *J* = 5.2 Hz, H-6″), 9.15 (d, 1H, *J* = 8.4 Hz, H-4″), 9.51 (d, 1H, *J* = 1.3 Hz, H-2″); ^13^C (50 MHz, DMSO-d_6_) δ: 46.0 (t), 55.4 (q), 58.1 (q), 70.6 (t), 102.5 (d), 108.7 (s), 111.9 (d), 112.2 (d), 115.8 (d), 125.1 (s), 127.3 (d), 131.1 (d), 131.7 (s), 133.0 (s), 139.5 (d), 140.8 (d), 141.6 (d), 147.6 (s), 155.0 (s), 164.1 (s). Anal. Calcd for: C_20_H_20_BrN_3_O_2_S: C, 53.82; H, 4.52; N, 9.41%. Found: C, 53.65; H, 4.47; N, 9.66%.

Tert-Butyl 5-Bromo-3-[4-(Pyridin-3-yl)-1,3-Thiazol-2-yl]-1H-Indole-1-Carboxylate Hydrobromide (**2g**)

Yellow solid; yield: 78%; mp: 185–186 °C; IR cm^−1^: 1749 (CO), 3421 (NH^+^); ^1^H NMR (200 MHz, DMSO-*d*_6_) δ: 1.68 (s, 9H, CH_3_); 7.63 (dd, 1H, *J* = 8.9, 2.0 Hz, H-6′), 8.01–8.13 (m, 2H, H-5″ and H-7′), 8.45 (s, 1H, 2′), 8.57 (d, 1H, *J* = 2.0 Hz, H-4′), 8.60 (s, 1H, H-5), 8.89 (d, 1H, *J* = 5.5 Hz, H-6″), 8.99 (d, 1H, *J* = 8.3 Hz, H-4″), 9.46 (d, 1H, *J* = 1.8 Hz, H-2″); ^13^C (50 MHz, DMSO-*d*_6_) δ: 27.6 (3 x q), 85.5 (s), 113.4 (s), 116.5 (d), 116.8 (s), 118.2 (d), 123.5 (d), 127.1 (d), 127.6 (d), 128.0 (s), 128.1 (d), 132.2 (s), 133.7 (s), 140.3 (d), 140.9 (d), 141.9 (d), 148.1 (s), 148.5 (s), 161.3 (s). Anal. Calcd for: C_21_H_19_Br_2_N_3_O_2_S: C, 46.95; H, 3.56; N, 7.82%. Found: C, 47.18; H, 3.41; N, 7.56%.

5-Bromo-1-Methyl-3-[4-(Pyridin-3-yl)-1,3-Thiazol-2-yl]-1H-Indole Hydrobromide (**2h**)

Orange solid; yield: 94%; mp: 279–280 °C; IR cm^−1^: 3420 (NH^+^); ^1^H NMR (200 MHz, DMSO-*d*_6_) δ: 3.89 (s, 3H, CH_3_), 7.41 (dd, 1H, *J* = 8.7, 1.9 Hz, H-6′), 7.56 (d, 1H, *J* = 8.7 Hz, H-7′), 8.18 (dd, 1H, *J* = 8.2, 5.6 Hz, H-5″), 8.31 (s, 1H, H-2′), 8.43 (d, 1H, *J* = 1.9 Hz, H-4′), 8.49 (s, 1H, H-5), 8.92 (d, 1H, *J* = 5.6 Hz, H-6″), 9.09 (d, 1H, *J* = 8.2 Hz, H-4″), 9.47 (d, 1H, *J* = 1.8 Hz, H-2″); ^13^C (50 MHz, DMSO-*d*_6_) δ: 33.2 (q), 108.4 (s), 112.9 (d), 114.1 (s), 116.2 (d), 122.7 (d), 125.2 (d), 125.9 (s), 127.3 (d), 132.7 (d), 132.8 (s), 135.8 (s), 139.6 (d), 141.0 (d), 141.4 (d), 147.8 (s), 163.4 (s). Anal. Calcd for: C_17_H_13_Br_2_N_3_S: C, 45.26; H, 2.90; N, 9.31%. Found: C, 45.44; H, 2.65; N, 9.47%.

5-Bromo-1-(2-Methoxyethyl)-3-[4-(Pyridin-3-yl)-1,3-Thiazol-2-yl]-1H-Indole Hydrobromide (**2i**)

Red solid; yield: 87%; mp: 196–197 °C; IR cm^−1^: 3427 (NH^+^); ^1^H NMR (200 MHz, DMSO-*d*_6_) δ: 3.23 (s, 3H, CH_3_), 3.72 (t, 2H, *J* = 5.0 Hz, CH_2_), 4.46 (t, 2H, *J* = 5.0 Hz, CH_2_), 7.42 (dd, 1H, *J* = 8.8, 2.0 Hz, H-6′), 7.65 (d, 1H, *J* = 8.8 Hz, H-7′), 8.18 (dd, 1H, *J* = 8.2, 5.6 Hz, H-5″), 8.32 (s, 1H, H-2′), 8.44 (d, 1H, *J* = 2.0 Hz, H-4′), 8.51 (s, 1H, H-5), 8.92 (d, 1H, *J* = 5.6 Hz, H-6″), 9.10 (d, 1H, *J* = 8.2 Hz, H-4″), 9.49 (d, 1H, *J* = 1.7 Hz, H-2″); ^13^C (50 MHz, DMSO-*d*_6_) δ: 46.0 (t), 58.1 (q), 70.6 (t), 108.7 (s), 113.2 (d), 114.0 (s), 116.3 (d), 122.7 (d), 125.2 (d), 126.0 (s), 127.2 (d), 132.2 (d), 132.7 (s), 135.5 (s), 139.8 (d), 141.2 (d), 141.3 (d), 148.0 (s), 163.3 (s). Anal. Calcd for: C_19_H_17_Br_2_N_3_OS: C, 46.08; H, 3.46; N, 8.48%. Found: C, 46.33; H, 3.60; N, 8.66%.

5-Fluoro-3-[4-(Pyridin-3-yl)-1,3-Thiazol-2-yl]-1H-Indole Hydrobromide (**2j**)

Yellow solid; yield: 90%; mp: 284–285 °C; IR cm^−1^: 3159 (NH), 3428 (NH^+^); ^1^H NMR (200 MHz, DMSO-*d*_6_) δ: 7.13 (td, 1H, *J* = 11.6, 9.3, 2.5 Hz, H-6′), 7.54 (dd, 1H, *J* = 9.3, 4.6 Hz, H-7′), 8.06–9.19 (m, 2H, H-4′ and H-5″), 8.34 (d, 1H, *J* = 2.9 Hz, H-2′), 8.48 (s, 1H, H-5), 8.90 (d, 1H, *J* = 4.8 Hz, H-6″), 9.14 (d, 1H, *J* = 8.4 Hz, H-4″), 9.54 (d, 1H, *J* = 1.7 Hz, H-2″), 12.02 (bs, 1H, NH); ^13^C (50 MHz, DMSO-*d*_6_): 105.4 (d, *J_C6′-F_* = 24.2 Hz), 110.0 (d, *J_C’7a-F_* = 4.6 Hz), 110.9 (d, *J_C4′-F_* = 26.2 Hz), 113.5 (d, *J_C7′-F_* = 10.2 Hz), 116.2 (d), 124.4 (d, *J_C3′a-F_* = 10.9 Hz), 127.4 (d), 129.4 (d), 133.0 (s), 133.2 (s), 139.4 (d), 140.6 (d), 141.9 (d), 147.6 (s), 158.1 (d, *J_C5′-F_* = 234 Hz), 164.1 (s). Anal. Calcd for: C_16_H_11_BrFN_3_S: C, 51.08; H, 2.95; N, 11.17%. Found: C, 51.36; H, 3.09; N, 11.41%.

5-Fluoro-1-Methyl-3-[4-(Pyridin-3-yl)-1,3-Thiazol-2-yl]-1H-Indole Hydrobromide (**2k**)

Orange solid; yield: 92%; mp: 277–278 °C; IR cm^−1^: 3420 (NH^+^); ^1^H NMR (200 MHz, DMSO-*d*_6_) δ: 3.89 (s, 3H, CH_3_), 7.13 (td, 1H, *J* = 11.7, 9.2, 2.6 Hz, H-6′), 7.60 (dd, 1H, *J* = 9.2, 4.4 Hz, H-7′), 8.05 (dd, 1H, *J* = 11.7, 2.6 Hz, H-4′), 8.16 (dd, 1H, *J* = 8.2, 5.6 Hz, H-5″), 8.32 (s, 1H, H-2′), 8.48 (s, 1H, H-5), 8.91 (d, 1H, *J* = 5.6 Hz, H-6″), 9.19 (d, 1H, *J* = 8.2 Hz, H-4″), 9.52 (d, 1H, *J* = 1.6 Hz, H-2″); ^13^C (50 MHz, DMSO-*d*_6_): 33.2 (q), 105.6 (d, *J_C6′-F_* = 24.9 Hz), 110.0 (d, *J_C’7a-F_* = 4.6 Hz), 110.9 (d, *J_C4′-F_* = 25.7 Hz), 112.1 (d, *J_C7′-F_* = 10.3 Hz), 115.9 (d), 124.7 (d, *J_C3′a-F_* = 11.0 Hz), 127.2 (d), 132.8 (s), 133.0 (d), 133.8 (s), 139.7 (d), 141.0 (d), 141.5 (d), 147.8 (s), 158.3 (d, *J_C5′-F_* = 236 Hz), 163.6 (s). Anal. Calcd for: C_17_H_13_BrFN_3_S: C, 52.32; H, 3.36; N, 10.77%. Found: C, 52.14; H, 3.48; N, 10.53%.

5-Fluoro-1-(2-Methoxyethyl)-3-[4-(Pyridin-3-yl)-1,3-Thiazol-2-yl]-1H-Indole Hydrobromide (**2l**)

Orange solid; yield: 88%; mp: 202 °C; IR cm^−1^: 3421 (NH^+^); ^1^H NMR (200 MHz, DMSO-*d*_6_) δ: 3.24 (s, 3H, CH_3_), 3.72 (t, 2H, *J* = 5.1 Hz, CH_2_), 4.46 (t, 2H, *J* = 5.1 Hz, CH_2_), 7.17 (td, 1H, *J* = 11.7, 9.2, 2.6 Hz, H-6′), 7.69 (dd, 1H, *J* = 9.2, 4.5 Hz, H-7′), 8.07 (dd, 1H, *J* = 11.7, 2.6 Hz, H-4′), 8.16 (dd, 1H, *J* = 8.2, 5.6 Hz, H-5″), 8.33 (s, 1H, H-2′), 8.50 (s, 1H, H-5), 8.91 (d, 1H, *J* = 5.6 Hz, H-6″), 9.14 (d, 1H, *J* = 8.2 Hz, H-4″), 9.54 (d, 1H, *J* = 1.7 Hz, H-2″); ^13^C (50 MHz, DMSO-*d*_6_): 46.1 (t), 58.1 (q), 70.6 (t), 105.6 (d, *J_C6′-F_* = 25.0 Hz), 109.2 (d, *J_C’7a-F_* = 4.7 Hz), 110.9 (d, *J_C4′-F_* = 26.3 Hz), 112.4 (d, *J_C7′-F_* = 9.8 Hz), 116.0 (d), 124.8 (d, *J_C3′a-F_* = 10.8 Hz), 127.2 (d), 132.5 (d), 132.8 (s), 133.4 (s), 139.9 (d), 141.2 (d), 141.4 (d), 147.9 (s), 162.1 (d, *J_C5′-F_* = 234 Hz), 163.6 (s). Anal. Calcd for: C_19_H_17_BrFN_3_OS: C, 52.54; H, 3.95; N, 9.67. Found: C, 52.23; H, 3.77; N, 9.81.

3-[4-(Pyridin-3-yl)-1,3-Thiazol-2-yl]-1H-Pyrrolo[2,3-b]Pyridine Hydrobromide (**2m**)

Yellow solid; yield: 95%; mp: 324–325 °C; IR cm^−1^: 3227 (NH), 3423 (NH^+^); ^1^H NMR (200 MHz, DMSO-*d*_6_) δ: 7.46 (dd, 1H, *J* = 7.9, 5.1 Hz, H-5′), 8.23 (dd, *J* = 8.2, 5.7 Hz, 1H, H-5″), 8.47–8.49 (m, 2H, H-2′ and H-6′), 8.63 (s, 1H, H-5), 8.94–8.95 (m, 2H, H-4′ and H-6″), 9.27 (d, 1H, *J* = 8.2 Hz, H-4″), 9.63 (d, 1H, *J* = 1.4 Hz, H-2″), 12.50 (bs, 1H, NH); ^13^C (50 MHz, DMSO-*d*_6_): 109.3 (s), 117.3 (2 x d), 118.4 (s), 127.5 (d), 128.8 (d), 132.3 (d), 132.9 (s), 139.2 (d), 140.5 (d), 141.3 (d), 142.4 (d), 145.4 (s), 147.7 (s), 165.0 (s). Anal. Calcd for: C_15_H_11_BrN_4_S: C, 50.15; H, 3.09; N, 15.60%. Found: C, 50.42; H, 3.20; N, 15.83%.

1-Methyl-3-[4-(Pyridin-3-yl)-1,3-Thiazol-2-yl]-1H-Pyrrolo[2,3-b]Pyridine Hydrobromide (**2n**)

Yellow solid; yield: 99%; mp: 280–282 °C; IR cm^−1^: 3425 (NH^+^); ^1^H NMR (200 MHz, DMSO-*d*_6_) δ: 3.92 (s, 3H, CH_3_), 7.37 (dd, 1H, *J* = 7.9, 4.8 Hz, H-5′), 8.21 (dd, *J* = 8.2, 5.7 Hz, 1H, H-5″), 8.44 (dd, 1H, *J* = 4.8, 1.5 Hz, H-6′), 8.49 (s, 1H, H-2′), 8.58 (s, 1H, H-5), 8.78 (dd, 1H, *J* = 7.9, 1.5 Hz, H-4′), 8.95 (d, 1H, *J* = 5.7 Hz, H-6″), 9.23 (d, 1H, *J* = 8.2 Hz, H-4″), 9.59 (d, 1H, *J* = 1.7 Hz, H-2”); ^13^C (50 MHz, DMSO-*d*_6_): 31.6 (q), 107.6 (s), 116.9 (d), 117.3 (s), 117.5 (d), 127.5 (d), 130.2 (d), 131.6 (d), 132.9 (s), 139.1 (d), 140.4 (d), 142.3 (d), 143.2 (d), 146.8 (s), 147.6 (s), and 163.2 (s). Anal. Calcd for: C_16_H_13_BrN_4_S: C, 51.48; H, 3.51; N, 15.01%. Found: C, 51.76; H, 3.72; N, 14.92%.

#### 3.1.5. General Procedure for the Synthesis of Thiazole Compounds (**1o–q**)

To a suspension of appropriate thiazole **1a,d,g** (0.38 mL) in DCM (5 mL) trifluoroacetic acid (7.0 mmol, 0.54 mL) was added, and the mixture was heated under reflux for 24 h. After cooling, the mixture was neutralized with saturated aqueous sodium hydrogen carbonate solution and extracted with dichloromethane (3 x 20 mL). The organic phases were dried (Na_2_SO_4_), evaporated under reduced pressure, and the obtained residue was recrystallized with ethanol to afford the desidered thiazoles **1o–q**.

3-[4-(Thiophen-3-yl)-1,3-THIAZOL-2-yl]-1H-indole (**1o**)

Yellow solid; yield: 95%; mp: 181–182 °C; IR cm^−1^: 3216 (NH); ^1^H NMR (200 MHz, DMSO-*d*_6_) δ: 7.22–7.27 (m, 2H, H-5′ and H-6′), 7.50–7.54 (m, 1H, H-7′), 7.64–7.72 (m, 2H, H-4″ and H-5″), 7.81 (s, 1H, H-5), 8.06 (dd, 1H, *J* = 2.8, 1.4 Hz, H-2″), 8.19 (d, 1H, *J* = 2.9 Hz, H-2′), 8.29–8.33 (-1H, H-4′); 11.86 (bs, 1H, NH); ^13^C (50 MHz, DMSO-*d*_6_) δ: 109.8 (s), 110.1 (d), 112.3 (d), 120.3 (d), 121.0 (d), 122.4 (d), 122.5 (d), 124.2 (s), 126.3 (d), 127.1 (d), 127.2 (d), 135.9 (s), 136.5 (s), 149.4 (s), 163.0 (s). Anal. Calcd for C_15_H_10_N_2_S_2_: C, 63.80; H, 3.57; N, 9.92%. Found: C, 63.57; H, 3.45; N, 10.22%.

5-Methoxy-3-[4-(Thiophen-3-yl)-1,3-THIAZOL-2-yl]-1H-indole (**1p**)

Orange solid; yield: 98%; mp: 183–184 °C; IR cm^−1^: 3264 (NH); ^1^H NMR (200 MHz, DMSO-*d*_6_) δ: 3.87 (s, 3H, CH_3_), 6.89 (dd, 1H, *J* = 8.8, 2.5 Hz, H-6′), 7.40 (d, 1H, *J* = 8.8 Hz, H-7′), 7.63–7.75 (m, 2H, H-4″ and H-5″), 7.75 (s, 1H, H-5), 7.84 (d, 1H, *J* = 2.5 Hz, H-4′), 8.00 (dd, 1H, *J* = 2.8, 1.4 Hz, H-2″), 8.07 (d, 1H, *J* = 2.9 Hz, H-2′), 11.63 (bs, 1H, NH); ^13^C (50 MHz, DMSO-*d*_6_) δ: 55.2 (q), 102.1 (d), 109.6 (d), 110.1 (s), 112.4 (d), 112.9 (d), 122.0 (d), 124.8 (s), 126.2 (d), 127.0 (d), 127.2 (d), 131.5 (s), 135.5 (s), 150.0 (s), 154.6 (s), 163.0 (s). Anal. Calcd for C_16_H_12_N_2_OS_2_: C, 61.51; H, 3.87; N, 8.97%. Found: C, 61.30; H, 3.68; N, 9.15%.

5-Bromo-3-[4-(Thiophen-3-yl)-1,3-Thiazol-2-yl]-1H-Indole (**1q**)

Yellow solid; yield: 72%; mp: 164–165 °C; IR cm^−1^: 3269 (NH); ^1^H NMR (200 MHz, DMSO-*d*_6_) δ: 7.37 (dd, 1H, *J* = 8.9, 1.9 Hz, H-6′), 7.49 (d, 1H, *J* = 8.9 Hz, H-7′), 7.68–7.69 (m, 2H, H-4″ and H-5″), 7.80 (s, 1H, H-5), 8.00 (t, 1H, *J* = 2.1 Hz, H-2″), 8.22 (d, 1H, *J* = 2.8 Hz, H-2′), 8.61 (d, 1H, *J* = 1.9 Hz, H-4′), 11.99 (bs, 1H, NH); ^13^C (50 MHz, DMSO-*d*_6_) δ: 110.0 (s), 110.2 (d), 113.4 (s), 114.2 (d), 122.0 (d), 122.7 (d), 125.0 (d), 126.0 (s), 126.2 (d), 127.1 (d), 128.2 (d), 135.3 (s), 136.5 (s), 150.3 (s), 162.2 (s). Anal. Calcd for C_15_H_9_BrN_2_S_2_: C, 49.87; H, 2.51; N, 7.75%. Found: C, 49.66; H, 2.42; N, 7.57%.

#### 3.1.6. General Procedure for the Synthesis of Thiazole Derivatives (**2o–q**)

To a suspension of appropriate thiazole **2a,d,g** (0.38 mL) in DCM (5 mL), trifluoroacetic acid (7.0 mmol, 0.54 mL) was added, and the mixture was heated under reflux for 24 h. After cooling, the solvent was evaporated under reduced pressure, and the obtained residue was recrystallized with ethanol to afford the desidered thiazoles **2-q**.

3-[4-(Pyridin-3-yl)-1,3-Thiazol-2-yl]-1H-Indole Hydrobromide (2o)

Yellow solid; yield: 72%; mp: 204–205 °C; IR cm^−1^: 3558 (NH), 3426 (NH^+^); ^1^H NMR (200 MHz, DMSO-*d*_6_) δ: 7.24–7.31 (m, 2H, H-5′ and H-6′), 7.49–7.57 (m, 1H, H-7′), 8.05 (dd, 1H, *J* = 8.1, 5.4 Hz, H-5″), 8.25 (d, 1H, *J* = 2.9 Hz, H-2′), 8.35–8.39 (m, 1H, H-4′), 8.41 (s, 1H, H-5), 8.85 (dd, 1H, *J* = 5.4, 1.8 Hz, H-6″), 8.99–9.05 (m, 1H, H-4″), 9.49 (d, 1H, *J* = 1.8 Hz, H-2″), 11.90 (bs, 1H, NH); ^13^C (50 MHz, DMSO-*d*_6_) δ: 109.9 (s), 112.3 (d), 115.8 (d), 120.5 (d), 121.0 (d), 122.6 (d), 124.1 (s), 127.2 (d), 127.6 (d), 132.9 (s), 136.6 (s), 139.9 (d), 141.1 (d), 141.4 (d), 147.8 (s), 164.4 (s). Anal. Calcd for: C_16_H_12_BrN_3_S: C, 53.64; H, 3.38; N, 11.73%. Found: C, 53.43; H, 3.24; N, 11.96%.

5-Methoxy-3-[4-(Pyridin-3-yl)-1,3-Thiazol-2-yl]-1H-Indole Hydrobromide (**2p**)

Orange solid; yield: 95%; mp: 228–229 °C; IR cm^−1^: 3313 (NH), 3421 (NH^+^); ^1^H NMR (200 MHz, DMSO-*d*_6_) δ: 3.89 (s, 3H, CH_3_), 6.72 (dd, 1H, *J* = 8.8, 2.5 Hz, H-6′), 7.43 (d, 1H, *J* = 8.8 Hz, H-7′), 7.84 (d, 1H, *J* = 2.5 Hz, H-4′), 8.09 (dd, 1H, *J* = 8.1, 5.5 Hz, H-5″), 8.19 (d, 1H, *J* = 2.9 Hz, H-2′), 8.42 (s, 1H, H-5), 8.87 (d, 1H, *J* = 5.5 Hz, H-6″), 9.06 (d, 1H, *J* = 8.1 Hz, H-4″), 9.50 (d, 1H, *J* = 2.0 Hz, H-2″), 11.79 (d, 1H, *J* = 2.9 Hz, NH); ^13^C (50 MHz, DMSO-*d*_6_) δ: 55.3 (q), 102.3 (d), 109.7 (s), 112.4 (d), 113.0 (d), 115.1 (d), 124.7 (s), 126.9 (d), 127.9 (d), 131.6 (s), 132.6 (s), 140.4 (d), 140.6 (d), 141.9 (d), 148.0 (s), 154.8 (s), 164.5 (s). Anal. Calcd for: C_17_H_14_BrN_3_OS: C, 52.59; H, 3.63; N, 10.82%. Found: C, 52.35; H, 3.51; N, 11.08%.

5-Bromo-3-[4-(Pyridin-3-yl)-1,3-Thiazol-2-yl]-1H-Indole Hydrobromide (**2q**)

Yellow solid; yield: 86%; mp: 245–246 °C; IR cm^−1^: 3193 (NH), 3422 (NH^+^); ^1^H NMR (200MHz, DMSO-*d*_6_) δ: 7.40 (dd, 1H, *J* = 8.6, 2.0 Hz, H-6′), 7.51 (d, 1H, *J* = 8.6 Hz, H-7′), 8.00 (dd, 1H, *J* = 8.1, 5.4 Hz, H-5″), 8.31 (d, 1H, *J* = 2.9 Hz, H-2′), 8.39 (s, 1H, H-5), 8.49 (d, 1H, *J* = 2.0 Hz, H-4′), 8.82 (d, 1H, *J* = 5.4 Hz, H-6″), 8.91 (d, 1H, *J* = 8.1 Hz, H-4″), 9.44 (d, 1H, *J* = 1.6 Hz, H-2″), 12.09 (bs, 1H, NH); ^13^C (50 MHz, DMSO-*d*_6_) δ: 109.5 (s), 113.6 (s), 114.4 (d), 115.6 (d), 122.6 (d), 125.2 (d), 125.8 (s), 126.7 (d), 129.0 (d), 132.4 (s), 135.3 (s), 140.1 (d), 140.9 (d), 142.3 (d), 148.4 (s), 163.7 (s). Anal. Calcd for: C_16_H_11_Br_2_N_3_S: C, 43.96; H, 2.54; N, 9.61%. Found: C, 43.68; H, 2.68; N, 9.42%.

### 3.2. Biology

#### 3.2.1. Minimum Inhibitory Concentrations (MICs) Determination

MICs were determined by using a microdilution method as recommended by CLSI for bacteria that grow aerobically (CLSI) (Clinical and Laboratory Standards Institute. Methods for Dilution Antimicrobial Susceptibility Tests for Bacteria That Grow Aerobically; Approved Standard—Ninth Edition. CLSI document M07-A9 (ISBN 1-56238-783-9 [Print]; ISBN 1-56238-784-7 [Electronic])) and Tryptic Soy Broth (TSB) (VWR International, Leuven, Belgium) as medium. It starts with the preparation of the bacterial suspension by diluting the bacteria grown at 37 °C for 24 h on Tryptic Soy Agar (TSA) (VWR International, LLC, Leuven, Belgium ) in 5 mL of 0.9% NaCl to obtain a culture suspension whose optical density (O.D.) at 570 nm is of about 0.190 (corresponding to 10^6^ colony-forming units CFU/mL); a further dilution 1:20 is always carried out in physiological solution. Serial dilutions 1:2 of the substance to be tested are performed, starting from the highest concentration, in culture medium (TSB); then, 10 μL of the prepared inoculum was added to each well containing substance solution. A positive growth control (test bacterial strain in the medium without inhibitor), a substance control (only the substance solution without inoculum), and a negative control (only the medium without inoculum), to check respectively the bacterial growth, the absorbance of substance at the highest concentration, and the sterility of medium, were also included in the assay. Thus, the plate prepared is incubated at 37 °C for 24 h, MICs were read by a microplate spectrophotometer (GloMax^®^-Multi Detection System, Promega Italia s.r.l, Milan, Italy) as the lowest concentration of sample whose O.D. at 570 nm is comparable to O.D. values of negative control wells.

#### 3.2.2. Inhibition of Biofilm Formation (Crystal Violet Method)

Bacterial strains were incubated in test tubes with TSB (5 mL) containing 2% *w/v* glucose at 37 °C for 24 h. After that, the bacterial suspensions were diluted to achieve a turbidity equivalent to a 0.5 McFarland standard. The diluted suspension (2.5 μL) was added to each well of a single cell culture polystyrene sterile, flat-bottom 96-well plate filled with TSB (200 μL) with 2% *w/v* glucose. Sub-MIC concentration values of all compounds were directly added to the wells to reach concentrations ranging from 100 to 0.1 μM to assess BIC_50_ values—that is, the concentration at which the percentage of inhibition of biofilm formation is equal to 50%. Plates were incubated at 37 °C for 24 h. After biofilm growth, the content of each well was removed, wells were washed twice with sterile NaCl 0.9% and stained with 200 μL of 0.1% *w/v* crystal violet solution for 15 min at 37 °C. Excess solution was removed, and the plate was washed twice, using tap water. A volume of 200 μL of ethanol was added to each stained well to solubilize the dye. Optical density (O.D.) was read at 600 nm using a microplate reader (GloMax^®^-Multi Detection System, Promega Italia s.r.l, Milan, Italy).

The experiments were run at least in triplicates, and three independent experiments were performed.

The percentage of inhibition was calculated through the formula:

% Inhibition = (OD growth − OD sample/OD growth control) × 100.(1)

#### 3.2.3. Antibiofilm Activity Against Pre-Formed Biofilm

A suspension of bacteria (0.5 McFarland standard) was obtained using the procedure described above for the inhibition of biofilm formation test. First, 2.5 mL of suspension was added to each well of a 96-well plate containing TSB (200 μL) with 2% *w/v* glucose. After the growth of a biofilm (24 h old), the content of each well was removed; then, wells were washed up twice with sterile PBS (Phosphate Buffered Saline) and filled with fresh TSB medium (200 μL). After that, different concentrations of compounds were added starting from a concentration equal or greater than the MIC obtained against the planktonic form of tested strains using TSB as the medium. The microtiter plate was sealed and incubated at 37 °C for further 24 h. The content of each well was removed, wells were washed twice with sterile PBS (100 mL to each well) and the 96-well plate was placed at 37 °C for 1 h before staining with a 0.1% *w/v* crystal violet solution. After 30 min, plates were washed with tap water to remove any excess stain.

Biofilm formation was determined by solubilizing crystal violet as above described, and the absorbance was read at 540 nm using a microplate reader (Glomax Multidetection System Promega, Promega Italia s.r.l, Milan, Italy). The percentages of inhibition were calculated with the above-reported. Each assay was performed in triplicate and repeated at least twice.

#### 3.2.4. Inhibition of Biofilm Formation (Viable Plate Count)

Compounds **1l**, **2b**, **2c**, **2i**, and **2k**, which exhibited the highest potency in inhibiting *S.aureus* ATCC 25923 biofilm formation by the crystal violet method, were tested against the same strain by using a viable plate counts method [22]. Briefly, a suspension of the tested strain was obtained as described in Section 3.2.2. Polystyrene flat-bottom 24-well plates were filled with 2 mL of TSB with 2% *w/v* glucose; then, we added 25 μL of bacterial suspension and sub-MIC concentrations (10; 5; 1; 0.1 μg/mL) of the above-mentioned compounds and incubated them for 24 h at 37 °C. After that time, the wells were washed 3 times with 1 mL of sterile NaCl (0.9% *v/v* solution), and the surface of each well was scraped 3 times. The inocula were put in test tubes with 10 mL of NaCl (0.9% *v/v* solution) and sonicated (ultrasonic nominal power equal to 215 kHz) for 2 min. Eight serial dilutions 1:10 were prepared and 100 μL aliquots of each dilution were plated onto tryptic soy agar (TSA). Then, petri dishes were incubated at 37 °C and CFU/mL were counted after 24 h. Each assay was performed in triplicate and repeated at least twice. Activity was expressed as log reduction with respect to the not treated growth control.

#### 3.2.5. Statistical Analysis

Mean values, standard deviation (SD), and significance testing (*p*-value) were calculated on a PC with the computer program, Microsoft Excel 2019 (Microsoft Corporation, Redmond, WA, USA).

## 4. Conclusions

With the aim to identify novel therapeutic approaches targeting antibiotic resistance mechanisms, two new series of 3-[4-(thiophen-3-yl)-1,3-thiazol-2-yl]-1*H*-indoles **1a-n** and 3-[4-(pyridin-3-yl)-1,3-thiazol-2-yl]-1*H*-indole hydrobromides 2a-n were efficiently synthesized and evaluated for their anti-biofilm activity. For almost all the new compounds, a marked selectivity toward the staphylococcal strains has been observed. In particular, compounds **1l** and **2i** showed the highest potency against *S. aureus* ATCC 25923, eliciting BIC_50_ values of 3.9 and 1.0 µM, respectively. Whereas, the 3-[4-(thiophen-3-yl)-1,3-thiazol-2-yl]-1*H*-indole **1a** and 3-[4-(pyridin-3-yl)-1,3-thiazol-2-yl]-1*H*-indole **2m** proved to be good inhibitors of *P. aeruginosa* biofilm formation with BIC_50_ values of 14.9 and 5.5 µM, respectively. 

Among the novel approaches evaluated in response to the emergence of the antibiotic resistance, the anti-virulence strategy is considered one of the most encouraging [23]. Disarming the bacteria from their pathogenicity tools, as the biofilm formation, was found to be more beneficial than interfering with their growth. In this scenario, the new thiazole derivatives **1l**, **2b**, **2c**, **2i**, and **2m**, which proved to be able to interfere with the biofilm formation, without affecting the microbial vital processes, can be considered promising lead compounds for the development of new anti-virulence agents usable for the treatment of biofilm-associated infections or for the prophylaxis of implant surgery.

## Figures and Tables

**Table 1:**
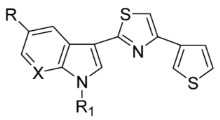
Substituted 3-[4-(thiophen-3-yl)-1,3-thiazol-2-yl]-1*H*-indoles **1a–q**.

Compd	R	R_1_	X	Compd	R	R_1_	X
**1a**	H	Boc	CH	1j	F	H	CH
**1b**	H	Me	CH	1k	F	Me	CH
**1c**	H	CH_2_CH_2_OMe	CH	1l	F	CH_2_CH_2_OMe	CH
**1d**	OMe	Boc	CH	1m	H	H	N
**1e**	OMe	Me	CH	1n	H	Me	N
**1f**	OMe	CH_2_CH_2_OMe	CH	1o	H	H	CH
**1g**	Br	Boc	CH	1p	OMe	H	CH
**1h**	Br	Me	CH	1q	Br	H	CH
**1i**	Br	CH_2_CH_2_OMe	CH				

**Table 2:**
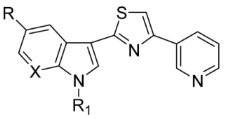
Substituted 3-[4-(pyridin-3-yl)-1,3-thiazol-2-yl]-1*H*-indole hydrobromides **2a–q**.

Compd	R	R_1_	X	Compd	R	R_1_	X
**2a**	H	Boc	CH	2j	F	H	CH
**2b**	H	Me	CH	2k	F	Me	CH
**2c**	H	CH_2_CH_2_OMe	CH	2l	F	CH_2_CH_2_OMe	CH
**2d**	OMe	Boc	CH	2m	H	H	N
**2e**	OMe	Me	CH	2n	H	Me	N
**2f**	OMe	CH_2_CH_2_OMe	CH	2o	H	H	CH
**2g**	Br	Boc	CH	2p	OMe	H	CH
**2h**	Br	Me	CH	2q	Br	H	CH
**2i**	Br	CH_2_CH_2_OMe	CH				

**Table 3:** Inhibition of biofilm formation, BIC_50_, µg/mL and µM.

Comp	*S.aureus* ATCC 25923		*S. aureus* ATCC 6538		*P. aeruginosa* ATCC 15442	
	µg/mL	µM	µg/mL	µM	µg/mL	µM
**1a**	n.s.	n.s.	14.5 ± 0.9	37.9	5.7 ± 0.5	14.9
**1b**	n.s.	n.s.	n.s.	n.s.	14.7 ± 1.2	49.5
**1c**	5.7 ± 0.7	16.7	6.6 ± 0.5	19.3	n.s.	n.s.
**1d**	n.s.	n.s.	5.7 ± 0.3	13.8	25.7 ± 1.7	62.2
**1e**	n.s.	n.s.	38.1 ± 1.8	116.7	11.2 ± 0.8	34.3
**1f**	18.5 ± 0.9	49.9	n.s.	n.s.	n.s.	n.s.
**1g**	8.1 ± 0.4	17.5	12.7 ± 0.9	27.5	10.6 ± 0.9	22.9
**1h**	10.1 ± 0.1	26.9	n.s.	n.s.	n.s.	n.s.
**1i**	11.2 ± 0.8	26.7	2.04 ± 0.07	4.8	n.s.	n.s.
**1j**	n.s.	n.s.	19.1 ± 0.9	63.5	35.6 ± 1.3	118.5
**1k**	n.s.	n.s.	3.9 ± 0.1	12.4	n.s.	n.s.
**1l**	1.4 ± 0.09	3.9	4.6 ± 0.2	12.8	n.s.	n.s.
**1m**	n.s.	n.s.	3.7 ± 0.08	13.0	n.s.	n.s.
**1o**	n.s.	n.s.	21.8 ± 1.3	77.1	12.6 ± 0.7	44.6
**1p**	n.s.	n.s.	n.s.	n.s.	13.6 ± 1.1	43.5
**1q**	n.s.	n.s.	16.4 ± 1.2	45.4	8.1 ± 0.6	22.4
**2b**	2.6 ± 0.1	6.9	12.9 ± 0.9	34.6	n.s.	n.s.
**2c**	3.8 ± 0.2	9.1	14.4 ± 1.1	34.5	13.3 ± 0.5	31.9
**2e**	n.s.	n.s.	4.8 ± 0.2	11.9	n.s.	n.s.
**2h**	n.s.	n.s.	17.1 ± 0.9	37.9	n.s.	n.s.
**2i**	0.5 ± 0.008	1.0	10.4 ± 0.8	20.1	n.s.	n.s.
**2j**	5.6 ± 0.4	14.8	9.2 ± 0.7	24.4	n.s.	n.s.
**2k**	3.2 ± 0.2	8.1	3.0 ± 0.01	7.6	n.s.	n.s.
**2l**	6.9 ± 0.4	15.8	n.s.	n.s.	12.5 ± 1.5	28.7
**2m**	n.s.	n.s.	n.s.	n.s.	0.2 ± 0.007	5.5
**2o**	15.2 ± 1.2	42.4	6.9 ± 0.5	19.2	n.s.	n.s.
**2p**	n.s.	n.s.	9.8 ± 0.3	25.2	n.s.	n.s.

n.s.: not significant because lower than 20% of inhibition percentage at the screening concentration of 100 µg/mL. The averages from at least three independent experiments are reported with standard deviation (SD).

## Sample Availability

Samples of the compounds are available from the authors.
